# Comprehensive Oxidative Stress Profiling and Clinical Correlates in Spondyloarthritis: The Role of Glutathione Peroxidase and Modifiable Lifestyle Factors

**DOI:** 10.3390/jcm14217747

**Published:** 2025-10-31

**Authors:** Rim Dhahri, Insaf Fenniche, Ismail Dergaa, Halil İbrahim Ceylan, Nicola Luigi Bragazzi, Lobna Ben Ammar, Hiba Ben Ayed, Ba Afif, Chakib Mazigh, Imène Gharsallah

**Affiliations:** 1 Faculty of Medicine of Tunis, University of Tunis El Manar, Tunis 1001, Tunisia; rimdhahri@ymail.com (R.D.); insaffenniche@gmail.com (I.F.); lobna.b.ammar@gmail.com (L.B.A.); benayedhiba3@gmail.com (H.B.A.); imengharsallah2005@gmail.com (I.G.); 2 Rheumatology Department, Military Hospital of Tunis, Tunis 1006, Tunisia; 3 High Institute of Sport and Physical Education of Ksar Said, University of Manouba, Manouba 2010, Tunisia; phd.dergaa@gmail.com; 4 Physical Education of Sports Teaching Department, Faculty of Sports Sciences, Ataturk University, Erzurum 25240, Türkiye; 5 Laboratory for Industrial and Applied Mathematics (LIAM), Department of Mathematics and Statistics, York University, Toronto, ON M3J 1P3, Canada; 6 Faculty of Pharmacy of Monastir, University of Monastir, Monastir 5000, Tunisia; afifba173@gmail.com (B.A.); chkouba.mazigh@gmail.com (C.M.); 7 Biochemistry Department, Military Hospital of Tunis, Tunis 1008, Tunisia

**Keywords:** spondyloarthritis, oxidative stress biomarkers, glutathione peroxidase, mediterranean diet, anti-TNF therapy, clinical implementation, personalized medicine

## Abstract

**Background:** Oxidative stress represents a key pathogenic factor in spondyloarthritis (SpA), yet its comprehensive assessment remains underutilized in routine clinical practice. **Objectives:** We evaluated oxidative stress biomarker profiles in SpA patients to determine associations with disease activity, systemic inflammation, structural damage, lifestyle factors, and therapeutic responses for practical clinical implementation. **Methods:** This cross-sectional study included 101 patients meeting the Assessment of SpondyloArthritis International Society (ASAS) 2009 criteria. Oxidative stress assessment utilized a validated biomarker panel: copper, zinc, glutathione peroxidase (GPx), ceruloplasmin (Cp), transferrin (TF), haptoglobin (Hp), bilirubin (BR), and uric acid (UA). Clinical, radiological, lifestyle, and therapeutic data underwent systematic analysis. **Results:** Glutathione peroxidase activity was elevated in 82.1% of patients, establishing it as the most sensitive oxidative stress marker. Copper levels increased in 30.7% and zinc deficiency occurred in 36.4% of cases. Oxidative stress markers correlated significantly with inflammatory parameters (erythrocyte sedimentation rate [ESR], C-reactive protein [CRP], neutrophil-to-lymphocyte ratio [NLR], platelet-to-lymphocyte ratio [PLR], neutrophil-to-monocyte ratio [NMR], systemic immune-inflammation index [SII]) and disease activity scores (Bath Ankylosing Spondylitis Disease Activity Index [BASDAI], Ankylosing Spondylitis Disease Activity Score based on CRP [ASDAS-CRP], Disease Activity Score 44 [DAS44-CRP]). Higher oxidative stress was associated with a poorer quality of life, as indicated by elevated Ankylosing Spondylitis Quality of Life (ASQoL) scores. Physical activity and adherence to a Mediterranean diet were independently associated with better antioxidant capacity. Smoking and nonsteroidal anti-inflammatory drug (NSAID) use correlated with increased oxidative burden. Anti-tumor necrosis factor alpha (anti-TNFα) therapy was associated with reduced levels of oxidative stress. Structural damage, particularly cervical spine involvement, correlated with heightened oxidative stress. **Conclusions:** This comprehensive evaluation reveals significant clinical correlations between oxidative stress and multiple disease domains in SpA. Modifiable lifestyle factors and therapeutic interventions have a significant impact on the redox balance. These findings establish practical targets for personalized management. The integration of oxidative stress assessment into routine practice could enhance disease monitoring and inform the development of antioxidant-based therapeutic strategies.

## 1. Introduction

Spondyloarthritis (SpA) encompasses a group of chronic inflammatory conditions that affect 0.20% to 1.61% of the global population [1]. These diseases primarily impact young adults with notable male predominance [2]. The conditions include axial spondyloarthritis, peripheral arthritis, and various extra-articular manifestations. Each imposes a substantial burden on patients’ function and quality of life [3]. Economic consequences extend beyond healthcare costs to include reduced productivity, disability, and long-term care needs [4]. Therefore, SpA represents a significant public health challenge requiring innovative management approaches.

Current understanding reveals SpA as a multifactorial disease involving complex interactions between genetic predisposition, environmental triggers, mechanical stress, and immune dysfunction [5]. These factors collectively drive chronic inflammation and progressive tissue damage. Recent evidence highlights the critical role of oxidative stress in disease pathogenesis. Oxidative stress reflects an imbalance between the production of reactive oxygen species and the antioxidant defense mechanisms [6]. This imbalance contributes to cellular damage through lipid peroxidation, protein carbonylation, and DNA modifications [7]. These processes perpetuate inflammatory cascades and accelerate tissue destruction [8].

The relationship between oxidative stress and inflammatory pathways in SpA involves a complex interplay, where excessive production of reactive oxygen species disrupts the redox balance, leading to molecular damage and the activation of key inflammatory signaling pathways, such as nuclear factor kappa B (NF-κB) and tumor necrosis factor-alpha (TNF-α). These factors regulate cytokine expression and cellular responses to oxidative damage [9]. Growing evidence suggests oxidative imbalance functions as both a consequence of inflammation and a potential therapeutic target [10]. Interventions targeting oxidative stress may reduce inflammation and prevent structural damage [11]. Studies investigating inflammatory arthritis have demonstrated that anti-TNF therapy significantly reduces oxidative stress markers, including 4-hydroxy-2-nonenal and thiobarbituric acid-reactive substances [12,13]. These findings suggest therapeutic modulation of redox balance represents a viable treatment strategy.

However, several critical knowledge gaps persist regarding the application of oxidative stress in the clinical management of SpA. Previous investigations have primarily examined isolated biomarkers rather than comprehensive oxidative stress profiles, which limits our understanding of redox patterns across different disease phenotypes and therapeutic contexts [14,15]. The relationship between oxidative stress and modifiable lifestyle factors remains inadequately characterized, despite potential implications for personalized treatment strategies [16,17]. Current assessment tools for extra-articular manifestations in SpA require further validation and correlation with systemic inflammatory markers [18]. The impact of biologic therapies on comorbidity outcomes and oxidative stress parameters needs systematic evaluation as well [19]. Furthermore, the association between inflammatory markers and functional disability across different rheumatic conditions suggests common pathways that warrant investigation in SpA populations [20]. Finally, current clinical practice lacks standardized approaches for assessing oxidative stress, which prevents the translation of research findings into practical patient care applications.

Given these research gaps, our study aimed to (i) provide comprehensive oxidative stress profiling in a well-characterized SpA cohort with systematic evaluation of clinical correlations across multiple disease domains; (ii) examine relationships between oxidative biomarkers and inflammatory activity, structural damage, functional impairment, and quality of life measures; and (iii) evaluate modifiable lifestyle factors including physical activity patterns and Mediterranean diet adherence as potential therapeutic targets while establishing practical frameworks for incorporating oxidative stress assessment into routine SpA management.

## 2. Methods

### 2.1. Study Design and Participants

This monocentric cross-sectional study was conducted from November 2024 to May 2025. Patients were consecutively recruited using a non-probability sampling method, including all eligible individuals during the study period. We included 101 patients with axial or peripheral SpA meeting the 2009 Assessment of SpondyloArthritis International Society (ASAS) classification criteria [21]. All participants were aged 18 years or older. Exclusion criteria included uncontrolled cardiovascular or metabolic diseases, medications that affect oxidative stress or inflammatory markers, such as high-dose antioxidant supplements and high-dose corticosteroids [22] (daily prednisone equivalent dose exceeding 30 mg [23]), systemic diseases, and pregnancy. Patients on conventional synthetic or biologic disease-modifying antirheumatic drugs (DMARDs) were not excluded. Both active and inactive, treated or biologic-naïve patients were eligible.

### 2.2. Ethical Approval

This study was conducted in accordance with the Declaration of Helsinki and received approval from the Ethics Committee of the Military Hospital of Tunis (approval number 93/2024/CLPP/Military Hospital of Tunisia). All participants provided written informed consent in both French and Arabic after receiving a detailed explanation of the study’s objectives, methods, and participation requirements.

### 2.3. Data Collection

#### Demographic and Clinical Assessment

A standardized survey form was used to collect demographic and clinical data through patient interviews and physical examinations conducted by the same investigator. Data included sex, age, and comorbidities defined according to European League Against Rheumatism (EULAR) criteria [24]. Lifestyle factors assessed included smoking, quantified by pack-years, alcohol consumption, physical activity measured using the International Physical Activity Questionnaire (IPAQ) short form [25], and adherence to the Mediterranean diet, evaluated using the Chrono Med Diet Score (CMDS) [26].

Disease-related data included age at onset, disease duration, human leukocyte antigen B27 (HLA-B27) status, SpA phenotype, extra-muscular manifestations, disease severity according to the Haute Autorité de Santé criteria [27], and current treatments. Disease activity assessment utilized the Bath Ankylosing Spondylitis Disease Activity Index (BASDAI) [28], Ankylosing Spondylitis Disease Activity Score based on C-reactive protein (ASDAS-CRP) [29], and Disease Activity Score based on 44-joint count and C-reactive protein (DAS44-CRP) [30]. Functional impact and quality of life evaluation employed the Bath Ankylosing Spondylitis Functional Index (BASFI) [28] and Ankylosing Spondylitis Quality of Life (ASQoL) [31]. Axial mobility measurement used the Bath Ankylosing Spondylitis Metrology Index (BASMI) [32].

### 2.4. Imaging Assessment

Imaging evaluation included the Modified Stoke Ankylosing Spondylitis Spine Score (mSASSS) [33], Bath Ankylosing Spondylitis Radiology Index (BASRI) [34], and Spondyloarthritis Research Consortium of Canada (SPARCC) scores [35].

### 2.5. Laboratory Investigations

#### 2.5.1. Sample Collection and Laboratory Procedures

Venous blood samples were collected from all participants in the morning (between 8:00 and 10:00 AM) after an overnight fast of at least 10 h.

Blood was drawn into plain and EDTA tubes, immediately centrifuged at 3000 rpm for 10 min at 4 °C. The resulting serum and plasma aliquots were then stored at −80 °C until analysis.

All assays were performed within 15 days of sample collection to minimize degradation and ensure analytical reliability.

#### 2.5.2. Inflammatory Markers

The standard inflammatory assessment included the erythrocyte sedimentation rate (ESR), C-reactive protein (CRP), and a complete blood count. Additional calculated ratios comprised the neutrophil-to-lymphocyte ratio (NLR), platelet-to-lymphocyte ratio (PLR), neutrophil-to-monocyte ratio (NMR), monocyte-to-lymphocyte ratio (MLR), and the systemic immune-inflammation index (SII) [36].

#### 2.5.3. Oxidative Stress Markers

Biomarker selection reflected the most robust and frequently reported parameters identified in recent systematic reviews and meta-analyses [37]. Selection prioritized cost-effectiveness and technical availability within our laboratory. Measured markers included copper, zinc, glutathione peroxidase (GPx), ceruloplasmin (Cp), transferrin (TF), haptoglobin (Hp), bilirubin (BR), and uric acid (UA). The copper/zinc ratio was calculated for each participant.

Copper quantification was performed using inductively coupled plasma mass spectrometry (ICP-MS) on an Agilent 7800 or 7900 system (Agilent Technologies, Santa Clara, CA, USA), with a reference range of 10–20 µmol/L. Zinc determination was conducted using atomic absorption spectroscopy, based on the absorption of the 213.9 nm spectral line emitted by a zinc hollow cathode lamp, with a standard range of 11–24 µmol/L. GPx activity was assessed using a colorimetric enzymatic assay on a Cobas analyzer employing Randox reagents, with reference values ranging from 4171–10,881 U/L. Cp, TF, and Hp concentrations were measured using an immunoturbidimetric assay on the DXC 700 AU Beckman Coulter analyzer (Beckman Coulter, Brea, CA, USA), with reference ranges of 0.20–0.60 g/L, 2.0–3.6 g/L, and 0.3–2.0 g/L, respectively. BR was quantified using the colorimetric diazo method, and UA was measured by the colorimetric enzymatic uricase method on the same analyzer. Reference values were <17 µmol/L for BR and 150–360 µmol/L for UA. All assays were performed according to the manufacturer’s instructions, and internal quality controls were applied to ensure analytical reliability.

#### 2.5.4. Oxidative Stress Definition

High oxidative stress was defined according to a composite biomarker profile. Increased copper, Cp, and copper/zinc ratio levels indicated elevated oxidative stress [38]. GPx activity typically increases during early oxidative stress as a protective response [39]. During prolonged or severe oxidative stress, GPx activity may decrease due to enzyme exhaustion. Therefore, both high and low GPx activity were interpreted as markers of oxidative stress, depending on the context. Zinc, TF, Hp, BR, and UA levels usually decrease under oxidative stress, reflecting their consumption in neutralizing reactive oxygen species [40,41,42].

#### 2.5.5. Statistical Analysis

Statistical analyses were performed using SPSS software, version 20 (IBM Corp., Armonk, NY, USA). Quantitative variables were summarized as mean ± standard deviation for normally distributed data and as medians with interquartile ranges (IQRs) for non-normally distributed data. In contrast, qualitative variables were expressed as counts and percentages. Before performing comparisons, the normality of distributions was assessed using Kolmogorov–Smirnov tests, and the homogeneity of variances was verified using Levene’s test. For group comparisons, parametric tests (Student’s *t*-test) were applied to variables with normal distributions and equal variances. In contrast, non-parametric tests (Mann–Whitney *U* test) were used when these assumptions were not met. Categorical variables were compared using the Pearson chi-square test or Fisher’s exact test, as appropriate. Correlations between continuous variables were evaluated using Pearson’s or Spearman’s correlation coefficients according to the data distribution. To identify factors independently associated with oxidative stress biomarkers, a multiple linear regression analysis was performed using the stepwise method. Statistical significance was defined as *p* < 0.05.

#### 2.5.6. Sample Size Calculation

The minimum sample size was estimated based on the number of SpA patients consulting in our department during the study period (*n* = 130).

Assuming a 95% confidence level (z = 1.96), a 5% margin of error (E = 0.05), and an estimated proportion (*p*) = 0.5 to ensure maximum sample size, we applied Cochran’s formula with finite population correction [43]:

$$n=\frac{N\times z^{2}\times p\times (1-p)}{E^{2}\times \left(N-1\right)+z^{2}\times p\times (1-p)}$$

The calculated minimum required sample size was 97 patients.

## 3. Results

### 3.1. Baseline Characteristics

The study population consisted of 101 patients with SpA. Table 1 summarizes baseline characteristics, comorbidities, and lifestyle factors. The mean age was 40.5 ± 11.4 years, with 40% of patients falling within the 30–40 years age range. The male-to-female ratio was 4.9. At least one comorbidity was present in 57.4% of patients (*n* = 58). Regarding lifestyle factors, 50% of patients (*n* = 49) reported moderate physical activity. Twenty-seven patients (26.7%) adhered to the principles of the Mediterranean diet.

### 3.2. Disease Characteristics

Table 2 presents clinical, biochemical, radiological characteristics, and ongoing treatments. Disease duration exceeded 10 years in 17.8% of patients (*n* = 18). At least one extra-articular manifestation occurred in 27.7% of patients (*n* = 28). Elevated ESR and CRP levels were found in 54.7% (*n* = 52) and 41.7% (*n* = 40) of patients, respectively. BASDAI ≥ 4 occurred in 12.8% of patients (*n* = 13). A BASFI score of ≥ 4 was observed in 51.5% (*n* = 52). BASMI ≥ 4 occurred in 21.7% (*n* = 22). A BASRI score ≥ 3 in the cervical or lumbar spine was noted in 27.7% of patients (*n* = 28).

### 3.3. Oxidative Stress Parameters

Table 3 shows oxidative stress biomarker levels. Elevated serum copper levels (>20 µmol/L) occurred in 30.69% of patients (*n* = 31). Increased GPx activity (>10,881 U/L) was detected in 82.10% of cases (*n* = 78). No patient had elevated Cp levels (greater than 0.60 g/L). Decreased Cp levels (<0.20 g/L) occurred in 5% of cases (*n* = 5). Zinc deficiency (<11 µmol/L) was identified in 36.63% of cases (*n* = 37). TF deficiency (<2 g/L) occurred in 8.24% of patients (*n* = 8). Elevated TF levels (>3.6 g/L) were reported in one patient. Elevated Hp levels (>2 g/L) were found in 34% of patients (*n* = 34). High BR levels (>17 µmol/L) occurred in 2% (*n* = 2). Increased UA levels (>360 µmol/L) were reported in 12.9% (*n* = 13)—no patients presented with decreased bilirubin or uric acid levels.

### 3.4. Clinical Correlations of Oxidative Stress Biomarkers

Oxidative stress status correlated significantly with older age (copper: r = 0.206, *p* = 0.039) and male sex, reflected by higher copper (*p* = 0.049) and copper/zinc ratio (*p* = 0.001) levels, and lower zinc (*p* = 0.010) and UA (*p* = 0.002) levels compared to those in female patients. Female patients exhibited significantly higher Cp levels (*p* = 0.026) and lower TF levels (*p* = 0.003), consistent with physiologically higher levels of these markers in females.

Antioxidant capacity appeared better preserved in patients without depression, reflected by significantly higher GPx activity compared to patients with depression (23,924.4 ± 20,914 vs. 13,773.1 ± 2330.7; *p* = 0.049). Oxidative stress was observed in patients with combined axial and peripheral involvement, reflected by significantly lower UA levels (*p* = 0.009), and in those with isolated peripheral involvement, who had decreased Hp concentrations (*p* = 0.028).

Oxidative stress was associated with extra-articular manifestations, particularly ocular involvement (GPx: 34,119.1 ± 27,504.9 vs. 20,000.1 ± 17,315.5; *p* = 0.021). Functional impairment correlated with elevated copper and Cp levels, which correlated positively with ASQoL scores (copper: r = 0.234, *p* = 0.020; Cp: r = 0.232, *p* = 0.021). No statistically significant differences were found between oxidative stress markers and disease severity or BASFI scores.

### 3.5. Lifestyle Factors and Oxidative Stress

Table 4 shows no significant associations between oxidative stress biomarkers and smoking status, alcohol consumption, or Mediterranean diet adherence in univariate analysis. However, physically active patients exhibited a better antioxidant status, with significantly higher GPx activity (*p* = 0.014) and BR levels (*p* = 0.017) compared to inactive patients.

### 3.6. Correlations with Inflammatory Parameters and Disease Activity

Table 5 demonstrates correlations between oxidative stress and increased inflammatory parameters, as well as higher disease activity scores. Serum copper, copper/zinc ratio, and Cp correlated positively with ESR, CRP, NLR, PLR, SII, and ASDAS-CRP. Zinc and BR correlated negatively with MLR, and with CRP, PLR, and ASDAS-CRP, respectively. GPx activity correlated negatively with CRP.

Hp, a positive acute-phase protein that typically decreases during oxidative stress, was positively correlated with ESR, CRP, NLR, PLR, NMR, SII, BASDAI, ASDAS-CRP, and DAS44-CRP in our study.

### 3.7. Radiological Correlations

Table 6 presents correlations between oxidative stress biomarkers and structural damage. Low UA concentrations correlated negatively with BASRI scores in the cervical spine (r = −0.236; *p* = 0.043) and hips (r = −0.222; *p* = 0.032). Hp correlated positively with BASRI sacroiliac joint score (r = 0.214; *p* = 0.037). No other significant correlations were observed between oxidative stress markers and radiological scores.

### 3.8. Treatment Effects on Oxidative Stress

Table 7 shows that patients receiving non-steroidal anti-inflammatory drugs (NSAIDs) exhibited increased oxidative stress, reflected by significantly lower TF (*p* = 0.002) and BR (*p* = 0.042) levels compared to those not taking NSAIDs. No significant differences in oxidative stress markers were observed between patients treated with anti-TNFα agents, conventional synthetic DMARDs (csDMARDs), or corticosteroids and patients not receiving these treatments in univariate analysis.

### 3.9. Multivariate Analysis

Table 8 presents the results of multiple linear regression, identifying factors independently associated with increased oxidative stress in patients with SpA. Male sex, smoking, dyslipidemia, depression, uveitis, cardiac involvement, elevated CRP, ASDAS-CRP, BASDAI, and ASQoL scores, cervical BASRI score, and NSAID and csDMARD use were significantly associated with higher oxidative stress levels.

Higher CMDS was independently associated with increased GPx concentrations (β = −1284.34; 95% CI: −3050.69 to −482.02; *p* = 0.046). Anti-TNF therapy was linked to higher BR levels (β = 1.51; 95% CI: 0.62 to 3.64; *p* = 0.049). These findings suggest lower consumption of these antioxidants and potentially reduced oxidative stress state.

Higher BASDAI scores were associated with reduced oxidative stress in some markers. Body mass index (BMI) and physical activity scores correlated positively with increased UA levels.

## 4. Discussion

Our study aimed to assess oxidative stress profiles in patients with SpA and determine correlations with clinical manifestations, lifestyle factors, biological and radiological findings, and therapeutic responses. The primary finding revealed GPx enzymatic activity as the most frequently elevated parameter, occurring in 82.1% of patients. This was followed by increased serum copper concentrations in 30.7% and zinc deficiency in 36.4% of patients. TF deficiency was less common (8.2%). No patient exhibited elevated Cp levels or decreased Hp levels. These findings establish GPx as the most sensitive marker of oxidative stress in SpA.

GPx enzymatic activity appeared to decline in patients with more advanced oxidative imbalance, suggesting potential exhaustion of antioxidant defenses. Hp concentrations in our cohort seemed to reflect primarily inflammatory activity rather than direct antioxidant consumption. Our univariate analysis revealed oxidative stress associations with older age, male sex, combined axial and peripheral SpA, isolated peripheral involvement, uveitis, functional impairment, elevated inflammatory biomarkers, higher disease activity, cervical spine involvement, and NSAID use. Conversely, antioxidant capacity appeared better preserved in patients without depression and those who were physically active.

Multivariate analysis additionally identified smoking, cardiac involvement, and csDMARD use as independently associated with increased oxidative stress. Patients with higher adherence to the Mediterranean diet and those treated with anti-TNFα agents exhibited reduced oxidative stress levels.

### 4.1. Impact of Lifestyle Factors on Oxidative Stress

Cigarette smoking is a well-established source of free radicals and a contributor to oxidative stress [9]. Physical activity enhances vascular health and antioxidant defenses [16]. The Mediterranean diet is recognized for its antioxidant and anti-inflammatory benefits [44]. These factors appear to modulate oxidative stress in SpA.

Univariate analysis showed no significant differences in oxidative stress markers between smokers and non-smokers or between alcohol consumers and non-consumers. However, multivariate analysis revealed a negative association between smoking and TF levels, suggesting increased oxidative stress in smokers.

Physically active patients exhibited significantly higher GPx activity and BR concentrations compared to inactive patients (GPx: 25,220 ± 21,721 vs. 14,413 ± 7493 U/L, *p* = 0.014; BR: 9.6 ± 3.6 vs. 7.6 ± 3.4 µmol/L, *p* = 0.017). Univariate analysis found no association between Mediterranean diet adherence and oxidative stress markers. However, multivariate analysis demonstrated that higher CMDS were independently associated with increased GPx activity, indicating better-preserved antioxidant capacity among patients following this diet. Previous studies have not specifically investigated the influence of these modifiable lifestyle factors on oxidative stress in SpA.

### 4.2. Inflammation and Disease Activity Correlations

Several oxidative stress biomarkers showed significant correlations with inflammatory and disease activity parameters. This suggests heightened inflammation and increased disease activity associated with elevated oxidative stress in SpA patients. Our findings align with previous reports. Aiginger et al. demonstrated positive correlations between copper, Cp, and ESR in patients with SpA [45]. Oriente et al. observed positive associations between these markers and ESR in psoriatic arthritis [46]. Svenson et al. reported a strong inverse correlation between zinc and ESR in SpA patients (r = −0.57, *p* < 0.01) [47].

Chung et al. found an inverse correlation between glutathione peroxidase-3 and CRP in ANCA-associated vasculitis (r = −0.261, *p* = 0.028), though no significant association was observed with ESR [48]. Wang et al. reported that serum BR levels were negatively correlated with CRP (r = −0.4187, *p* < 0.001), indicating that consumption of both GPx and BR occurs during inflammatory states [49]. Wang et al. showed SpA patients with high disease activity (BASDAI ≥ 4) had lower erythrocyte GPx levels compared to those with BASDAI < 4 (19.09 U/g vs. 20.50 U/g), though this difference was not statistically significant [50].

### 4.3. Discussion of Radiological Correlations

Few studies have examined relationships between oxidative stress markers and structural damage in SpA. Lai et al. reported serum UA was significantly associated with radiographic axial SpA with an odds ratio of 1.06 (95% CI: 1.03–1.10; *p* < 0.001) [51]. In our study, UA concentrations correlated negatively with BASRI-cervical and BASRI-hip scores, suggesting that oxidative stress may be linked to structural involvement.

### 4.4. Functional Impairment Correlations

Increased oxidative stress was associated with ASQoL scores in our study. Cai et al. reported a positive association between serum UA levels and Short Form 36 (SF-36) scores in SpA patients, where higher scores indicate better perceived health status [52]. Previous studies have linked mood disorders with altered UA levels, attributed to potential effects of adenosine concentration and receptor signaling on anxiety and depression [53,54].

### 4.5. Broader Clinical Implications and Comorbidity Considerations

The relationship between oxidative stress and functional outcomes in our study aligns with current evidence from other inflammatory conditions. Recent investigations demonstrate significant associations between inflammatory markers and functional disability in chronic pain conditions [55]. This suggests common pathways linking inflammation, oxidative stress, and functional impairment. This connection supports our findings regarding correlations between ASQoL scores and oxidative stress markers. It emphasizes the importance of comprehensive assessment beyond traditional disease activity measures.

### 4.6. Therapeutic Implications

Several authors have explored antioxidant therapy as a potential adjunct or alternative treatment for chronic inflammatory rheumatic diseases. Currently, more than 700 clinical studies on antioxidant therapy are registered worldwide. In chronic inflammatory rheumatic diseases, zinc supplementation has been investigated, particularly in psoriatic arthritis. Clemmensen was among the first to report clinical improvement in patients with psoriatic arthritis treated with oral zinc sulfate [56].

Recent studies have emphasized the importance of maintaining zinc homeostasis in patients with psoriasis and psoriatic arthritis. They advocate for correcting copper and zinc imbalances as part of the therapeutic strategy [57,58]. Zinc’s immunomodulatory potential has been demonstrated in various settings, including the capacity to reduce inflammatory markers such as CRP [59,60]. BR has developed recognition as a promising antioxidant in nanomedicine. Bilirubin-based nanoparticles have been evaluated in preclinical models for the treatment of autoimmune diseases, including rheumatoid arthritis, psoriasis, and autoimmune encephalomyelitis [61].

A 2023 meta-analysis reviewed oxidative stress-targeted therapy in rheumatoid arthritis, encompassing 27 randomized controlled trials that investigated 16 different antioxidants. Only one randomized controlled trial failed to confirm the anti-inflammatory effect. The majority of studies reported reductions in CRP, ESR, TNF-α, interleukin-2 (IL-2), and IL-10 levels [62]. TNF-α inhibitors have been shown to modulate oxidative stress. Túnez et al. demonstrated that infliximab significantly reduced oxidative stress in patients with chronic inflammatory rheumatic diseases [63].

Solmaz et al. [64] reported lower total oxidative status and higher total antioxidant status in SpA patients treated with anti-TNF agents compared to those on NSAIDs. However, differences were not statistically significant [64]. Preclinical studies show activation of adenosine A2A receptors through purine metabolism has anti-inflammatory effects in both rheumatoid arthritis and SpA. This mechanism may partly explain the therapeutic effects of csDMARDs [53]. Prolonged NSAID therapy has been associated with increased oxidative stress [65].

In our cohort, univariate analysis revealed that patients receiving NSAIDs had higher oxidative stress. Multivariate analysis showed anti-TNF treatment was independently associated with a lower oxidative stress state. These findings align with the literature data.

### 4.7. Clinical Implementation Framework

The integration of oxidative stress assessment into routine SpA management requires practical consideration of several factors. Our findings suggest GPx activity represents the most sensitive and readily available biomarker for clinical implementation. The high prevalence of elevation (82.1%) combined with its correlation with disease activity and treatment response establishes it as a priority marker for routine assessment [66].

A cost-effectiveness analysis supports focusing on a limited panel of biomarkers rather than conducting a comprehensive assessment of all biomarkers. This makes GPx, copper, and zinc the most practical combination for clinical use. Laboratory standardization remains crucial for the clinical implementation of diagnostic tests. Our methodology, which utilizes readily available analytical techniques in most clinical laboratories, supports widespread adoption. Quality control measures and the establishment of reference ranges specific to SpA populations would enhance clinical utility. Integration with existing inflammatory marker assessment could provide comprehensive disease monitoring without a significant additional burden.

Patient education regarding modifiable lifestyle factors is a critical component of managing oxidative stress. Our findings support the use of structured counseling regarding Mediterranean diet adherence and physical activity as evidence-based interventions [67]. Smoking cessation programs warrant particular emphasis, given the strong association with oxidative stress in our multivariate analysis [68]. These interventions provide cost-effective approaches to enhancing redox balance, independent of pharmacological therapy.

Treatment monitoring applications include baseline assessment of oxidative stress before therapy initiation and periodic reassessment during treatment. Our data suggest that the effects of anti-TNF therapy on oxidative stress may precede changes in traditional inflammatory markers. This potentially offers an earlier indication of treatment response. This application requires validation in longitudinal studies with serial measurements.

### 4.8. Strengths and Limitations

This represents the first study providing a holistic evaluation of oxidative stress in SpA using validated quantitative methods. Our comprehensive approach offers a multidimensional view of redox imbalance across multiple disease domains. The systematic assessment of lifestyle factors and their correlation with oxidative stress markers provides novel data for clinical applications.

However, important limitations should be acknowledged. The monocentric design and cross-sectional nature of this study restrict the generalizability of the findings and limit causal inference. Local clinical practices, environmental exposures, and genetic backgrounds may differ, which could influence oxidative stress patterns. Future multicenter studies involving larger and more diverse populations from various geographic and ethnic backgrounds are warranted to enhance external validity and confirm these results.

The cross-sectional design also precludes the assessment of temporal relationships between oxidative stress biomarkers, disease activity, and treatment response. Longitudinal studies with repeated biomarker measurements would allow the evaluation of temporal dynamics and causal pathways linking redox imbalance to disease progression and therapeutic modulation, thereby strengthening causal interpretation.

Furthermore, the present study did not explore the influence of treatment duration or therapeutic adherence on oxidative stress levels, although therapies such as anti-TNF agents may modulate redox homeostasis. Incorporating detailed pharmacological histories and adherence metrics in future prospective studies would help clarify treatment-related effects on oxidative stress regulation.

To address these limitations, future research should prioritize large-scale, multicenter, and longitudinal designs integrating standardized treatment documentation and adherence assessment. Such studies will be essential to validate and extend the clinical applicability of our findings in SpA management.

## 5. Conclusions

Oxidative stress exhibits a close association with clinical, inflammatory, radiological, and lifestyle aspects of SpA. GPx activity develops as the most sensitive biomarker reflecting oxidative imbalance. Integrating oxidative stress assessment may enhance understanding of disease pathogenesis, facilitate personalized patient care, and open new avenues for redox-targeted therapeutic approaches in SpA.

The identification of modifiable lifestyle factors as significant determinants of oxidative stress provides actionable targets for patient counseling and the development of comprehensive care strategies. Future research should focus on longitudinal assessment of oxidative stress markers in response to therapeutic interventions. Validation of these biomarkers as predictors of treatment response and long-term outcomes is also needed.

### Practical Implementation Recommendations

The clinical practice integration of oxidative stress assessment should begin with the measurement of GPx activity as the primary screening tool. Copper and zinc assessment provides additional valuable information for a comprehensive evaluation. Patient counseling should emphasize adherence to the Mediterranean diet and regular physical activity as evidence-based interventions for improving redox balance.

Smoking cessation programs deserve particular attention, given strong associations with oxidative stress. Clinicians should consider assessing oxidative stress in patients with poor quality of life scores or an inadequate response to conventional therapy. Integration with existing inflammatory marker assessment can provide comprehensive disease monitoring without a significant additional burden.

## Figures and Tables

**Table 1:** Baseline Demographic, Clinical, and Lifestyle Characteristics of Patients with SpA. Data are expressed as mean ± standard deviation (SD) [range] or *n* (%). Reduced bone mineral density was assessed by dual-energy X-ray absorptiometry (DEXA) in 83 patients. Depression was evaluated in 98 patients using the Hospital Anxiety and Depression Scale (HAD). Cardiovascular (hypertension, dyslipidemia, myocardial infarction, stroke, acute limb ischemia), metabolic (diabetes), infectious, peptic ulcer, and neoplastic histories were obtained from patients’ medical records.

Parameter	Mean ± SD [Range] or *n* (%)
Age (years)	40.5 ± 11.4 [23–68]
Male gender, *n* (%)	84 (83.2%)
BMI (kg/m^2^)	26.07 ± 4.46 [17.24–37.72]
Reduced bone mineral density, *n* (%)	50% (*n* = 83 patients with DEXA)
Depression, *n* (%)	19 (18.8%) (screened in *n* = 98 by HAD questionnaire)
Hypertension, *n* (%)	9 (9%)
Dyslipidemia, *n* (%)	5 (5%)
Myocardial infarction, *n* (%)	4 (4%)
Diabetes, *n* (%)	3 (3%)
Infection, *n* (%)	3 (3%)
Peptic ulcer, *n* (%)	2 (2%)
Stroke, *n* (%)	1 (1%)
Acute limb ischemia, *n* (%)	1 (1%)
Neoplasia, *n* (%)	1 (1%)
Lifestyle factors	
Current smokers, *n* (%)	40 (39.6%)
Smoking exposure (PY)	5.95
Alcohol consumption	13 (12.87%)
Physical activity (IPAQ, MET-min/week)	2269.89 ± 3543.05 [0–22,848]
Mediterranean diet score (CMDS)	9.52 ± 4.84 [0–20]

*n*: number of subjects; BMI: Body Mass Index; PY: Pack-years; DEXA: Dual-energy X-ray absorptiometry; HAD: Hospital Anxiety and Depression Scale; IPAQ: International Physical Activity Questionnaire; MET: Metabolic Equivalent; CMDS: Chrono Med Diet Score.

**Table 2:** Clinical, Biochemical, Radiological Features, and Ongoing Treatments in SpA Patients. Data are expressed as mean ± standard deviation (SD) [range] or *n* (%). Extra-articular manifestations were defined as follows: anterior uveitis confirmed by ophthalmologic evaluation; pulmonary involvement defined by restrictive and/or obstructive ventilatory disorders on pulmonary function testing or interstitial/emphysematous lesions on chest imaging (X-ray or CT scan); cardiac involvement defined by valvular disease or conduction/rhythm disturbances based on clinical signs, electrocardiogram, and echocardiography; and renal involvement including AA amyloidosis, IgA nephropathy, or renal calculi not attributable to other causes, identified through renal function tests, 24 h proteinuria, renal ultrasound, CT urography, or kidney biopsy.

Parameter	Mean ± SD [Range] or *n* (%)
**Age at onset (years)**	35.1 ± 10.7 [18–67]
**Disease duration (years)**	5.7 ± 6.5 [0.5–31]
**SpA phenotypes, *n* (%)**	
Isolated axial SpA	58% (*n* = 59)
Axial and peripheral SpA	36.6% (*n* = 37)
Isolated peripheral SpA	5% (*n* = 5)
Inflammatory bowel disease	7.9% (*n* = 8)
Psoriasis	10.9% (*n* = 11)
**Extra-articular manifestations, *n* (%)**	
Uveitis	12% (*n* = 12)
Pulmonary involvement	13% (*n* = 13)
Renal involvement	11% (*n* = 11)
Cardiac involvement	5% (*n* = 5)
**Laboratory inflammatory markers**	
ESR (mm/h)	28.2 ± 25.1 [5–105]
CRP (mg/L)	10.16 ± 16.78 [2–91].
NLR	2.2 ± 1.3 [0.3–7.1]
PLR	129.9 ± 67.1 [50.6–443.6]
NMR	9.3 ± 6.2 [1.1–42.1]
MLR	0.26 ± 0.12 [0.05–0.9]
SII	660,278.5 ± 516,767.1 [51,789.4–2,539,466.6]
**Disease activity**	
BASDAI	2.39 ± 1.68 [0–8.10]
ASDAS-CRP	2.06 ± 1.16 [0–5.7]
DAS44-CRP	1.15 ± 0.68 [0.67–3.04].
**BASFI**	4.15 ± 2.29 [0–9.1]
**ASQoL**	7.93 ± 5.58 [0–18]
**BASMI**	2.76 ± 2.04 [0–9]
**Severe SpA, *n* (%)**	83.2% (*n* = 84)
**Imaging findings**	
BASRI score	6.91 ± 4.13 [0–16]
mSASSS	10.47 ± 14.45 [0–58]
SPARCC spine score	26.95 ± 25.91 [0–64]
SPARCC sacroiliac score	15.50 ± 21.40 [0–71]
**Treatment, *n* (%)**	
NSAIDs	60.4% (*n* = 61)
Corticosteroids	3% (*n* = 3)
CsDMARDs	20.8% (*n* = 21)
Anti-TNFα therapy	46.5% (*n* = 47)

*n*: number of subjects; SpA: Spondyloarthritis; ESR: Erythrocyte sedimentation rate; CRP: C-reactive protein; NLR: Neutrophil-to-lymphocyte ratio; PLR: Platelet-to-lymphocyte ratio; NMR: Neutrophil-to-monocyte ratio; MLR: Monocyte-to-lymphocyte ratio; SII: Systemic immune-inflammation index; BASDAI: Bath Ankylosing Spondylitis Disease Activity Index; ASDAS: Ankylosing Spondylitis Disease Activity Score; DAS44: Disease Activity Score 44-joint count; BASFI: Bath Ankylosing Spondylitis Functional Index; ASQoL: Ankylosing Spondylitis Quality of Life; BASMI: Bath Ankylosing Spondylitis Metrology Index; BASRI: Bath Ankylosing Spondylitis Radiology Index; mSASSS: Modified Stoke Ankylosing Spondylitis Spine Score; SPARCC: Spondyloarthritis Research Consortium of Canada; NSAIDs: Non-steroidal anti-inflammatory drugs; CsDMARDs: Conventional synthetic disease-modifying antirheumatic drugs; Anti-TNFα: Anti-tumor necrosis factor alpha.

**Table 3:** Levels of Oxidative Stress Biomarkers in Patients with SpA. Data are expressed as mean ± standard deviation (SD) [range].

Biomarker	Mean ± SD [Range]
**Cu (µmol/L)**	20.03 ± 5.06 [13–47]
**Zn (µmol/L)**	11.75 ± 2.17 [7.10–21.30]
**Cu/Zn ratio**	1.78 ± 0.67 [0.83–5.80]
**GPx (U/L)**	21,634.92 ± 19,127.18 [7259–95,350]
**Cp (g/L)**	0.26 ± 0.04 [0.17–0.49]
**TF (g/L)**	2.41 ± 0.42 [1.50–4.10]
**BR (μmol/L)**	8.90 ± 3.65 [2–18]
**Hp (g/L)**	1.75 ± 0.71 [0.30–3.53]
**UA (μmol/L)**	289.00 ± 64.72 [157–498]

Cu: Copper; Zn: Zinc; GPx: Glutathione peroxidase; Cp: Ceruloplasmin; TF: Transferrin; Hp: Haptoglobin; BR: Bilirubin; UA: Uric Acid; SD: Standard Deviation.

**Table 4:** Comparison of Oxidative Stress Biomarkers According to Lifestyle Factors in Patients with SpA.

Biomarker	Lifestyle Factors											
	Smoker			Alcohol Consumption			Physical Activity Level			Adherence to the Mediterranean Diet		
	Yes	No	*p*	Yes	No	*p*	Active	Inactive	*p*	Yes	No	*p*
Cu (µmol/L)	19.6 ± 4	20.3 ± 5.7	0.483	19.4 ± 3.6	20.1 ± 5.3	0.620	19.8 ± 5.5	20.2 ± 3.8	0.727	20.3 ± 6.9	19.8 ± 4.2	0.661
Zn (µmol/L)	12.2 ± 2.5	11.5 ± 1.9	0.086	12.4 ± 2	11.7 ± 2.2	0.227	11.6 ± 2	12.2 ± 2.7	0.275	11.7 ± 2.1	11.8 ± 2.3	0.867
Cu/Zn ratio	1.7 ± 0.5	1.9 ± 0.8	0.151	1.6 ± 0.3	1.8 ± 0.7	0.258	1.8 ± 0.7	1.7 ± 0.5	0.734	1.8 ± 0.9	1.7 ± 0.5	0.558
GPx (U/L)	21,663.9 ± 18,918	21,615.6 ± 19,433	0.990	17,141.5 ± 10,945.3	22,347.3 ± 20,076.4	0.365	25,220.2 ± 21,720.8	14,413.3 ± 7493.1	0.014	24,702 ± 21,339.6	21,164.2 ± 18,648.5	0.449
Cp (g/L)	0.27 ± 0.06	0.26 ± 0.04	0.257	0.3 ± 0	0.3 ± 0.1	0.739	0.27 ± 0.05	0.27 ± 0.03	0.506	0.27 ± 0.06	0.27 ± 0.04	0.871
TF (g/L)	2.3 ± 0.5	2.5 ± 0.4	0.080	2.4 ± 0.2	2.4 ± 0.5	0.658	2.4 ± 0.4	2.4 ± 0.4	0.737	2.4 ± 0.4	2.4 ± 0.5	0.697
BR (µmol/L)	8.3 ± 3.7	9.3 ± 3.6	0.193	9.6 ± 4.2	8.8 ± 3.6	0.493	9.6 ± 3.6	7.6 ± 3.4	0.017	9.3 ± 3.7	8.9 ± 3.6	0.668
Hp (g/L)	1.9 ± 0.7	1.6 ± 0.7	0.066	2 ± 0.6	1.7 ± 0.7	0.248	1.7 ± 0.7	1.8 ± 0.8	0.619	1.7 ± 0.7	1.8 ± 0.7	0.794
UA (µmol/L)	293.4 ± 54.2	286 ± 71.3	0.582	298.9 ± 69.5	287.5 ± 64.3	0.557	289.3 ± 63.7	290.6 ± 69.5	0.928	296.9 ± 56.7	287.1 ± 68	0.511

Cu: Copper; Zn: Zinc; Cu/Zn ratio: Copper-to-zinc ratio; GPx: Glutathione peroxidase; Cp: Ceruloplasmin; TF: Transferrin; BR: Bilirubin; Hp: Haptoglobin; UA: Uric acid; *p*: *p*-value.

**Table 5:** Correlations Between Oxidative Stress Biomarkers, Inflammatory Markers, and Disease Activity Scores in Patients with SpA.

Parameters	Cu	Zn	Cu/Zn Ratio	GPx	Cp	TF	BR	Hp	UA
Laboratory inflammatory markers									
ESR	0.521 ***	−0.091	0.469 ***	−0.099	0.516 ***	0.059	−0.133	0.371 ***	−0.110
CRP	0.518 ***	−0.042	0.432 ***	−0.211 *	0.541 ***	0.052	−0.355 **	0.637 ***	−0.180
NLR	0.262 **	−0.122	0.235 **	−0.125	0.339 **	−0.090	−0.169	0.355 ***	−0.055
PLR	0.308 **	−0.181	0.308 **	−0.151	0.334 **	−0.099	−0.221 **	0.352 ***	−0.152
MLR	0.071	−0.219 **	0.146	−0.179	0.081	−0.070	−0.158	0.178	−0.104
NMR	0.196	0.067	0.107	−0.015	0.259 **	−0.062	−0.117	0.210 **	−0.052
SII	0.336 **	−0.109	0.287 **	−0.146	0.404 ***	−0.077	−0.197	0.429 ***	−0.069
Disease activity									
BASDAI	0.154	−0.061	0.148	−0.073	0.171	−0.131	−0.170	0.228 **	−0.095
ASDAS-CRP	0.498 ***	−0.117	0.430 ***	−0.105	0.487 ***	−0.003	−0.207	0.441 ***	−0.031
DAS44-CRP	0.086	−0.124	0.129	−0.036	0.209	−0.053	0.013	0.185	0.026

Data are presented as the r value in Pearson or Spearman’s correlation test * *p* < 0.05, ** *p* < 0.01, *** *p* < 0.001. Cu: Copper; Zn: Zinc; Cu/Zn ratio: Copper-to-zinc ratio; GPx: Glutathione peroxidase; Cp: Ceruloplasmin; TF: Transferrin; BR: Bilirubin; Hp: Haptoglobin; UA: Uric acid; ESR: Erythrocyte sedimentation rate; CRP: C-reactive protein; NLR: Neutrophil-to-lymphocyte ratio; PLR: Platelet-to-lymphocyte ratio; MLR: Monocyte-to-lymphocyte ratio; NMR: Neutrophil-to-monocyte ratio; SII: Systemic immune-inflammation index; BASDAI: Bath Ankylosing Spondylitis Disease Activity Index; ASDAS-CRP: Ankylosing Spondylitis Disease Activity Score; DAS44-CRP: Disease Activity Score 44-joint count.

**Table 6:** Correlations Between Oxidative Stress Biomarkers and Radiological Scores in Patients with SpA.

Radiological Score	Cu	Zn	Cu/Zn Ratio	GPx	Cp	TF	BR	Hp	UA
**mSASSS**	0.098	0.168	−0.027	0.137	0.100	0.016	−0.049	0.177	0.053
**Cervical BASRI**	0.134	0.047	0.075	0.160	0.108	0.084	0.021	0.019	−0.236 *
**Lumbar BASRI**	0.078	0.142	−0.025	0.133	0.069	−0.067	−0.003	0.130	0.067
**Hip BASRI**	−0.052	0.139	−0.114	0.119	0.000	−0.031	0.144	−0.003	−0.222 *
**Sacroiliac BASRI**	0.157	0.066	0.063	0.119	0.182	0.011	−0.040	0.214 *	−0.001
**Total BASRI score**	0.100	0.158	−0.017	0.125	0.125	0.066	0.043	0.141	−0.109
**SPARCC Spine score**	0.316	0.289	−0.031	0.245	0.438	−0.163	−0.135	0.179	0.291
**SPARCC Sacroiliac score**	0.077	0.258	−0.093	−0.113	0.218	0.039	−0.101	0.311	0.016

Data are presented as the r value in Pearson or Spearman’s correlation test * *p* < 0.05. Cu: Copper; Zn: Zinc; Cu/Zn ratio: Copper-to-zinc ratio; GPx: Glutathione peroxidase; Cp: Ceruloplasmin; TF: Transferrin; BR: Bilirubin; Hp: Haptoglobin; UA: Uric acid; mSASSS: Modified Stoke Ankylosing Spondylitis Spine Score; BASRI: Bath Ankylosing Spondylitis Radiology Index; SPARCC: Spondyloarthritis Research Consortium of Canada.

**Table 7:** Oxidative Stress Biomarker Levels According to Ongoing Treatments in Patients with SpA.

	NSAIDs			Anti-TNFα Therapy			CsDMARDs			Corticosteroids		
	Yes	No	*p*	Yes	No	*p*	Yes	No	*p*	Yes	No	*p*
**Cu (µmol/L)**	20 ± 4	20.1 ± 6.4	0.923	19.9 ± 6.1	20.1 ± 4	0.849	20.9 ± 5.1	19.8 ± 5.1	0.408	21.3 ± 2.3	20 ± 5.1	0.656
**Zn (µmol/L)**	11.8 ± 2.2	11.6 ± 2.1	0.679	11.7 ± 2.1	11.8 ± 2.2	0.705	11.1 ± 2.3	11.9 ± 2.1	0.132	11.8 ± 4.6	11.8 ± 2.1	0.972
**Cu/Zn ratio**	1.8 ± 0.5	1.8 ± 0.9	0.655	1.8 ± 0.8	1.8 ± 0.5	0.788	2 ± 0.7	1.7 ± 0.7	0.145	2 ± 0.9	1.8 ± 0.7	0.544
**GPx (U/L)**	19,861.9 ± 15,932.9	24,294.5 ± 23,089.6	0.271	23,491.1 ± 21,205	19,892.4 ± 16,985.4	0.362	19,215 ± 17,612.7	22,280.3 ± 19,572.3	0.527	11,416 ± 1612.2	21,854.7 ± 19,273.2	0.448
**Cp (g/L)**	0.27 ± 0.04	0.27 ± 0.06	0.819	0.26 ± 0.06	0.27 ± 0.04	0.636	0.27 ± 0.05	0.27 ± 0.05	0.777	0.29 ± 0.01	0.27 ± 0.05	0.416
**TF (g/L)**	2.3 ± 0.3	2.6 ± 0.5	0.002	2.5 ± 0.4	2.4 ± 0.4	0.110	2.4 ± 0.6	2.4 ± 0.4	0.743	2.9 ± 1.1	2.4 ± 0.4	0.109
**BR (μmol/L)**	8.3 ± 3.6	9.8 ± 3.6	0.042	9.4 ± 3.8	8.5 ± 3.5	0.205	9.8 ± 3.8	8.7 ± 3.6	0.247	7.7 ± 4.6	8.9 ± 3.6	0.555
**Hp (g/L)**	1.8 ± 0.7	1.6 ± 0.7	0.143	1.7 ± 0.7	1.8 ± 0.8	0.261	1.8 ± 0.8	1.7 ± 0.7	0.914	2.3 ± 0.2	1.7 ± 0.7	0.213
**UA (μmol/L)**	291.2 ± 65.5	285.8 ± 64.2	0.688	283.6 ± 64	293.8 ± 65.6	0.437	275.6 ± 61	292.4 ± 65.6	0.301	245.7 ± 87.6	290.4 ± 64	0.241

NSAIDs: Non-steroidal anti-inflammatory drugs; Anti-TNFα: Anti–tumor necrosis factor alpha; CsDMARDs: Conventional synthetic disease-modifying antirheumatic drugs; Cu: Copper; Zn: Zinc; Cu/Zn ratio: Copper-to-zinc ratio; GPx: Glutathione peroxidase; Cp: Ceruloplasmin; TF: Transferrin; BR: Bilirubin; Hp: Haptoglobin; UA: Uric acid.

**Table 8:** Multiple linear regression studying factors associated with high oxidative stress in patients with SpA.

Biomarker	Variable	Standardized Coefficient (Beta)	Confidence Interval (95%)		*p*-Value
			Min	Max	
**Cu**	Male	2.706	0.392	5.804	0.049
	CRP	0.065	0.007	0.137	0.048
	BASDAI	−1.678	−2.703	−0.652	0.002
	ASDAS-CRP	3.288	1.643	4.932	0.000
	ASQol	0.365	0.005	0.725	0.047
**Zn**	Male	1.131	0.366	2.689	0.047
	Cardiac involvement	1.955	0.746	4.318	0.043
	ASDAS_CRP	−0.285	−0.744	−0.103	0.045
**Cu/Zn**	Osteoporosis	0.282	0.043	0.608	0.049
	ASDAS-CRP	0.233	0.034	0.432	0.023
	BASDAI	−0.109	−0.311	−0.013	0.048
	CsDMARDs	0.320	0.026	0.613	0.034
**GPx**	Depression	−5289.191	−8944.921	−1633.461	0.006
	Uveitis	19,094.811	9665.834	47,855.456	0.048
	BASDAI	6498.011	3216.553	16,212.575	0.047
	Mediterranean diet score (CMDS)	−1284.338	−3050.693	−482.016	0.046
**Cp**	CRP	0.001	0.001	0.002	0.001
	ASDAS-CRP	0.021	0.007	0.036	0.004
	BASDAI	−0.015	−0.023	−0.006	0.001
**TF**	Smoking	−0.194	−0.428	−0.040	0.044
	Dyslipidemia	−0.317	−0.608	−0.025	0.034
	Depression	0.557	0.219	0.896	0.002
	NSAID use	−0.232	−0.474	−0.011	0.046
**BR**	Osteoporosis	−2.720	−5.068	−0.372	0.024
	Depression	−1.956	−4.546	−0.634	0.044
	Hypertension	−2.443	−5.683	−0.797	0.037
	CRP	−0.119	−0.199	−0.039	0.004
	Anti-TNF therapy	1.514	0.615	3.643	0.049
**Hp**	Smoking	0.316	0.043	0.671	0.026
	CRP	0.027	0.016	0.038	0.000
	NSAID use	0.150	0.101	0.460	0.047
**AU**	Male	102.012	61.132	145.811	0.000
	BMI	6.997	3.082	10.913	0.001
	CRP	−1.029	−1.989	−0.069	0.036
	Cervical BASRI	−11.106	−20.084	−1.058	0.048
	Physical activity score (IPAQ)	−0.033	−0.067	−0.003	0.049

Cu: Copper; Zn: Zinc; GPx: Glutathione peroxidase; Cp: Ceruloplasmin; TF: Transferrin; Hp: Haptoglobin; BR: Bilirubin; UA: Uric Acid; CRP: C-reactive protein; BASDAI: Bath Ankylosing Spondylitis Disease Activity Index; ASDAS: Ankylosing Spondylitis Disease Activity Score; CsDMARDs: Conventional synthetic Disease-Modifying Anti-Rheumatic Drugs; ASQoL: Ankylosing Spondylitis Quality of Life questionnaire; CMDS: Chrono Med Diet Score; NSAID: Nonsteroidal Anti-Inflammatory Drug; Anti-TNF: Tumor Necrosis Factor inhibitor; BMI: Body Mass Index; BASRI: Bath Ankylosing Spondylitis Radiographic Index; IPAQ: International Physical Activity Questionnaire; Min: Minimum; Max: Maximum.

## Data Availability

The underlying data not included in this article are available from the corresponding author upon reasonable request.
